# Phototropic response features for different systematic groups of mesoplankton under adverse environmental conditions

**DOI:** 10.1002/ece3.8072

**Published:** 2021-11-16

**Authors:** Victor Dyomin, Yuri Morgalev, Igor Polovtsev, Alexandra Davydova, Sergey Morgalev, Nikolay Kirillov, Tamara Morgaleva, Alexey Olshukov

**Affiliations:** ^1^ Laboratory for Radiophysical and Optical Methods of Environmental Research National Research Tomsk State University Tomsk Russia; ^2^ Biotest‐Nano Center National Research Tomsk State University Tomsk Russia; ^3^ Laboratory of Environmental Remote Sensing V.E. Zuev Institute of Atmospheric Optics of Siberian Branch of the Russian Academy of Science Tomsk Russia

**Keywords:** bioindication, early diagnostics of ecosystem pollution, environmental monitoring, mesoplankton, phototropic response, submersible digital holographic camera

## Abstract

Current trends in the application of bioindication methods are related to the use of submersible tools that perform real‐time measurements directly in the studied aquatic environment. The methods based on the registration of changes in the behavioral responses of zooplankton, in particular *Crustaceans*, which make up the vast majority of the biomass in water areas, seem quite promising. However, the multispecies composition of natural planktonic biocenoses poses the need to consider the potential difference in the sensitivity of organisms to pollutants.

This paper describes laboratory studies of the phototropic response of plankton to attracting light. The studies were carried out on a model natural community that in equal amounts includes *Daphnia magna*, *Daphnia pulex,* and *Cyclops vicinus*, as well as on the monoculture groups of these species. The phototropic response was initiated by the attracting light with a wavelength of 532 nm close to the local maximum of the reflection spectrum of chlorella microalgae. Standard potassium bichromate was used as the model pollutant.

The largest phototropic response value is registered in the assemblage. The concentration growth rate of crustaceans in the illuminated volume was 4.5 ± 0.3 ind (L min)^−1^. Of the studied species, the phototropic response was mostly expressed in *Daphnia magna* (3.7 ± 0.4 ind (L min)^−1^), while in *Daphnia pulex,* it was reduced to 2.4 ± 0.2 ind (L min)^−1^, and in *Cyclops vicinus,* it was very small—0.16 ± 0.02 ind (L min)^−1^. This is caused by peculiar trophic behavior of phyto‐ and zoophages. The addition of a pollutant, namely potassium bichromate, caused a decrease in the concentration rate of crustaceans in the attracting light zone, while a dose‐dependent change in phototropic responses was observed in a group of species and the *Daphnia magna* assemblage.

The results of laboratory studies showed high potential of using the phototropic response of zooplankton to monitor the quality of its habitat thus ensuring the early diagnostics of water pollution. Besides, the paper shows the possibility of quantifying the phototropic response of zooplankton using submersible digital holographic cameras (DHC).

## Graphical abstract

This paper describes laboratory studies of the phototropic response of plankton to attracting light. The studies were carried out on a model natural community that in equal amounts includes *Daphnia magna*, *Daphnia pulex*, and *Cyclops vicinus*, as well as on the monoculture groups of these species. The phototropic response was initiated by the attracting light with a wavelength of 532 nm close to the local maximum of the reflection spectrum of chlorella microalgae. The results of laboratory studies showed high potential of using the phototropic response of zooplankton to monitor the quality of its habitat thus ensuring the early diagnostics of water pollution.

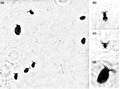

## 1 INTRODUCTION

The contamination of water bodies with small concentrations of pollutants at first may not have a visible toxic effect. Moreover, the violation of biological well‐being may not be detected during a single examination. But in case of chronic impact on the biota, this may lead to a shift in the ratio between species. This will inevitably cause changes in the quality of the ecosystem and potential disastrous reduction in the number of aboriginal species. Therefore, today there is an urgent need for prompt early control of pollution of natural water areas by microconcentration of pollutants (Sukharenko et al., 2017). Besides, early detection of pollutants is critical in hazardous areas such as nuclear stations, oil platforms, and gas pipelines.

Over the past two decades, quite a few studies have been devoted to water biotesting techniques based on the analysis of various features of aquatic organisms, namely survival registration (OECD, 2013, 2019), reproducibility, offspring quality, morphological parameter changes (OECD, 2013), physiological functions, and behavioral responses (Lechelt et al., 2000; Morgalev et al., 2015; Nikitin, 2014; Wang et al., 2019). The most promising are the methods of water biomonitoring using behavioral responses of local species of hydrobionts, and primarily bivalves (Sukharenko et al., 2017), branchiopoda (Carreño‐León et al., 2014) and copepoda (Lechelt et al., 2000; Pan et al., 2015, 2017; Ren et al., 2017), and fish (Ren et al., 2016). Daphnia are particularly interesting with regard to these methods. They filter a large amount of water by feeding on bacteria and algae contained in it, as a result of which the presence of harmful substances even at low concentrations causes significant changes in their state.

Behavioral response recording techniques are more sensitive than the methods that register mortality or growth and developmental inhibition. There are automated methods of continuous biological control that can generate an alarm signal based on recording physiological parameters and behavioral responses, such as speed and trajectory of swimming, frequency of swimming movements, etc. (Dodson et al., 1997; ISO, 2012; Lechelt et al., 2000). However, these methods are implemented by stationary flow devices, so the analyzed water samples shall be delivered to them, which significantly reduces the dynamics of monitoring. Besides, they use special laboratory types of daphnia aligned by sensitivity to model toxicants. Such disadvantages, including a limited set of test species, are typical for other devices of this type.

The current trend in world ocean monitoring is the use of submersible tools that perform real‐time measurements directly in the studied aquatic environment. The same approach should be applied to study the responses of biological species thus ensuring high representativeness of sampling and more reliable bioindication. The second important advantage here is the recording of responses of autochthonous organisms adapted to local changes in environmental factors. Therefore, the development of methods that record behavioral responses directly in the habitat is justified and promising.

Phototropism is particularly interesting among other behavioral responses. The phenomenon of phototaxis in hydrobionts has long been known. It is most clearly observed in diel vertical migration along the water column (Cousyn et al., 2001)—the largest biomass migration. Light‐dependent movement of microzooplankton reduces the biomass of phytoplankton at the surface (Moeller et al., 2019). With the illumination change, the aggregation of daphniae may be changed by toxicants, for example, by titanium oxide nanoparticles (Noss et al., 2013). On the one hand, the variability of diel vertical migration indicates the participation of the nervous system in the phototropic response: In environments where the influence of predators and other factors change over time, normal migration (nocturnal ascent) may be replaced by reverse migration (nocturnal descent) (Ohman, 1990). On the other hand, phototactic responses change when such psychotropic substances as diazepam, fluoxetine, and carbamazepine are introduced (Rivetti et al., 2016).

Therefore, it is possible to expect a significant change in the phototropic response when the nervous system of planktonic organisms changes as a result of pollutants in their habitat.

At the same time, there are contradictory data regarding the phototropic response: Thus, according to Simão et al. (2019), when faced with a sudden increase in light intensity, *Daphnia magna* show a photomotor reaction, period of hyperlocomotion, when animals try to escape from light to avoid predatory fish. At the same time, De Meester (1991) describes both swimming toward (positive phototaxis) and away from light (negative phototaxis).

Besides, most of such studies do not focus on the wavelength of stimulating light being only limited to the term “daylight.” Another significant disadvantage of most trajectory‐tracking studies is the small number of individuals: from 1 to 10 in different studies.

The laboratory studies on a large number of individuals, but not on single representatives of zooplankton, make it possible, firstly, to vary the spectrum and concentration of pollutants and, secondly, to obtain a statistically significant response assessment of hydrobionts. Besides, the use of natural autochthonous organisms (and not only their laboratory analogues) allows extrapolating the resulting patterns to subsequent field studies.

This requires special technical means that allow detecting and classifying indicator organisms in the monitoring mode, as well as monitoring the dynamics of their behavioral responses.

The operational oceanology utilizes a large number of devices used to study the properties of plankton using fluorimetric, nephelometric, and turbidimetric measurements (“SBE 19plus V2 SeaCAT Profiler CTD | Sea‐Bird Scientific—Overview | Sea‐Bird”, 2020). However, the registration using such equipment does not make it possible to perform differential bioindication, including the study of behavioral responses. There are several commercial submersible holographic cameras on the market that provide measurements of individual particles, in particular, LISST‐Holo (“Environmental Archives—Sequoia ScientificSequoia Scientific”, 2020; Ouillon, 2018) and Submersible Microscope (“HoloSea: Submersible Holographic Microscope—4Deep”, 2020; Rotermund & Samson, 2015). On the contrary, compared to photographic cameras (Cowen & Guigand, 2008; Lertvilai, 2020; Ohman et al., 2012) holographic cameras record information on all particles in volume per one exposure, which allows obtaining a focused image of each particle in the recorded volume from one hologram, identifying geometric parameters and classifying the type of each of the particles in the recorded volume, and using the time sequence of holograms to build a trajectory and study the motion pattern of each particle (Bochdansky et al., 2013; Graham et al., 2012; Nayak et al., 2018; Owen & Zozulya, 2000; Pfitsch et al., 2007; Sun et al., 2008; Talapatra et al., 2013). The studies (Dyomin, et al., 2019; Giering et al., 2020; Nayak et al., 2021) show the comparison of such cameras.

The equipment created at Tomsk State University (TSU) (digital holographic cameras and hydrobiological probes based on them) (Dyomin, Davydova, et al., 2020; Dyomin, Gribenyukov, Davydova, et al., 2019; Dyomin et al., 2018, 2019) also provides measurements of individual particles, but differs from known analogues in the possibility of photostimulation with attracting radiation causing a phototropic response of zooplankton (Dyomin, Davydova, Morgalev, Olshukov, et al., 2019). The advantages include both a large size of the controlled volume and thus obtained representativeness of data.

The purpose of this study was to assess the contribution of crustaceans of different species represented in natural freshwater populations to changing the phototropic responses of a group of hydrobionts under the action of pollutants.

## 2 MATERIALS AND METHODS

### 2.1 Experimental equipment and software

We used a digital holographic camera (DHC) developed by us to record the behavioral responses of zooplankton assemblage. The DHC allows registering a digital hologram of the entire studied volume of water with plankton per one exposure (laser pulse) and then restoring the image of this volume in a layers‐by‐layers mode. The software‐based DHC technology for digital hologram recording and processing allows automatically restoring the spatial distribution of particles in the studied volume (3D coordinates of each particle), determining the size, shape, speed, and direction of movement of each particle, and recognizing them (Dyomin, Davydova, et al., 2020; Dyomin, Davydova, Morgalev, Olshukov, et al., 2019; Dyomin, Gribenyukov, Davydova, et al., 2019; Dyomin et al., 2018; Dyomin, Polovtsev, Davydova, & Olshukov, 2019). Besides, it registers individuals with the minimum size of 100 μm at the resolution sufficient enough to identify plankton individuals. It is evident that in order to study the particle motion parameters, it is necessary to record a time sequence of digital volume holograms and reconstruct the video based on holographic data (Dyomin & Olshukov, 2012).

Thus, compared to existing analogues, the DHC provides the ability not only to measure dimensions and coordinates in the monitoring mode but also to classify plankton by species and assess the motor activity of plankton.

The device can be used in field studies up to a depth of 600 m (the depth of the information layer from the point of view of plankton presence). A hologram of 1‐L volume is recorded per one exposure. In the accumulation mode (e.g., when water passes through the measuring channel while moving the DHC), the studied volume may be increased to 15 L per second.

**FIGURE 1:**
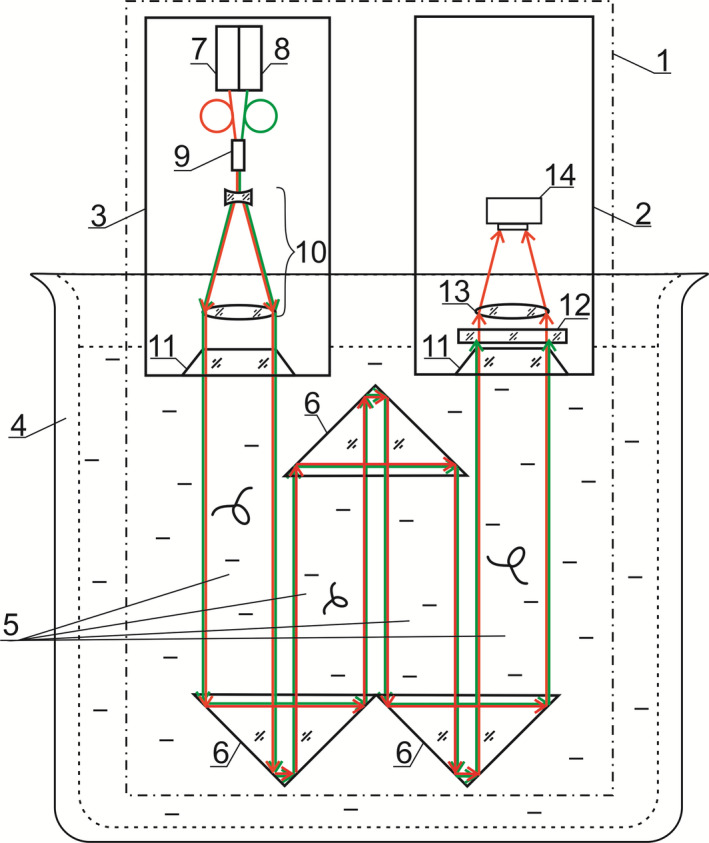
Laboratory setup: 1—DHC, 2—DHC recording unit, 3—DHC lighting unit, 4—laboratory water tank, 5—studied (working) volume formed by recording (red) and attracting (green) light beams, 6—mirror‐prism system to form the working volume, 7—semiconductor laser diode (*λ* = 650 nm), 8—semiconductor laser diode (*λ* = 532 nm), 9—fiber‐optic multiplexer (mixer), 10—beam expander, 11—portholes, 12—selective filter, 13—receiving lens, 14—CMOS camera

Figure 1 shows a scheme explaining the DHC operation and use in laboratory experiments.

The DHC base is composed of lighting (3) and recording (2) units located in sealed cases.

Light from the source 7 (*λ* = 650 nm) is formed by the beam expander (10) into a parallel beam with a diameter of 35 mm. Then, radiation passes through a porthole (11), the analyzed volume (5) with analyzed particles optically formed by the recording (lighting) beam and prisms (7) and gets into the recording unit (2) through a porthole (11). As a result, an interference pattern of the reference wave (part of the radiation that passed by the particles) and the object wave (part of the radiation scattered on the particles) is formed. The optical system (13) in the recording unit matches the size of the incoming beam with size of a CMOS camera (14). The camera (14) registers the mentioned interference pattern (which is then a registered digital hologram of the studied volume) and transmits it to the computer memory via a digital cable. Mathematical processing by computational algorithms (Dyomin, Gribenyukov, Davydova, et al., 2019) allows reconstructing the image of each particle (plankton individual) from one digital hologram, ensuring the spatial distribution of particles in the studied volume (3D coordinates of each particle), determining the size, shape, speed, and direction of movement of each particle, and recognizing them. Additional attracting radiation from the source (8) also passes through the volume with studied particles and is used for the photostimulation of zooplankton behavioral activity. A fiber‐optic multiplexer (9) is used to input this photostimulating green radiation of a semiconductor laser (*λ* = 532 nm) into the same DHC optical channel. In order to prevent the damage of the camera (14) matrix, radiation at a certain wavelength (*λ* = 532 nm) is absorbed by a selective filter (12).

This series of experiments utilized the recording red light (wavelength—650 nm, radiation power—20.3 mW) and attracting light (wavelength—532 nm, close to the local maximum of chlorella microalgae reflection spectrum, radiation power—9.6 mW). The radiation power is indicated at the output of the porthole.

In laboratory experiments for the phototropic response of plankton, the optical part of the DHC (1) was placed in a 90‐L laboratory water tank (4) filled with water containing plankton (Figure 1).

### 2.2 Studied organisms

The studies were carried out on the groups of individuals—Cladocera *Daphnia magna Straus* and *Daphnia pulex*, as well as Copepoda *Cyclops vicinus*. *Daphnia magna Straus* was obtained from the developers of the technique (Grigoriev & Shashkova, 2011). *Daphnia pulex* and *Cyclops vicinus* were taken from the natural population in freshwater reservoirs in Tomsk area and have been adapted to laboratory conditions for 8 months. Juveniles were collected by filtration through a mesh filter with a cell size of 2 × 2 mm. After classification, the individuals were moved away and kept in containers with artificial cultivation medium. Cultivation and experiments were carried out under the conditions recommended in the procedures (ISO, 2012): *t* = 22 ± 2℃, artificial lighting—500–1,000 lux, pH—7.0–8.5, control culture medium—fresh water, O_2_ content = 6 mg/L, photoperiod—12/12 hr.

The wild *Daphnia pulex* species were introduced into the laboratory culture according to the recommendations (Grigoriev & Shashkova, 2011). To introduce the *Cyclops vicinus* wild species into the laboratory culture, an introduction procedure was developed, including a feeding regime. A cocktail consisting of a decoction of lettuce leaves, a concentrate of single‐cell chlorella algae, and a suspension of *Paramecium caudata* infusories was used for feeding in the following proportion: 50 ml of “cocktail” containing 35 ml of lettuce leaves decoction at the concentration of 1 g/L, 10 ml of chlorella concentrate with an optical density of *D* = 0.450–0.500, and 5 ml of infusoria suspension at the concentration of 150 pcs/ml were added to 1 L of the cultivation medium.

To verify the stability of the culture prior to holographic registration, the sensitivity to the standard toxicant was assessed in accordance with (ISO, 2012).

The individuals of the same age (3 days) were used in the study. In the experiments with monoculture, 270 small crustaceans were placed in a 90‐L water tank, which corresponded to the concentration of 3,000 individuals per 1 m^3^. In case of mixed culture, 90 small crustaceans of each of the three considered species were used.

### 2.3 Experimental procedures

The experimental procedure for each zooplankton species or their mixture included the following sequence of operations (Table 1).

**TABLE 1:** Experimental procedures

	Time	Procedure
Day 1. Control	8:00 9:00 10:00	Placing crustaceans into a container. Feeding. Start of hologram hourly recording under control (without a toxicant).
Day 2. Experiment with a toxicant 0.06 mg/L	7:00 10:00	Completion of hourly hologram recording. Toxicant application. Start of hologram hourly registration in the conditions of the experiment.
Day 3. Control	7:00 8:00 9:00 10:00	Completion of hologram recording. Container cleaning. Change of cultivation medium. Adding a new group of crustaceans to the tank. Feeding. Start of hologram hourly recording under control.
Day 4. Experiment with a toxicant 0.12 mg/L	7:00 10:00	Completion of hologram hourly recording under control conditions. Toxicant application. Start of hologram hourly registration in the conditions of the experiment.
Day 5	7:00	Completion of hologram recording.

**FIGURE 2:**
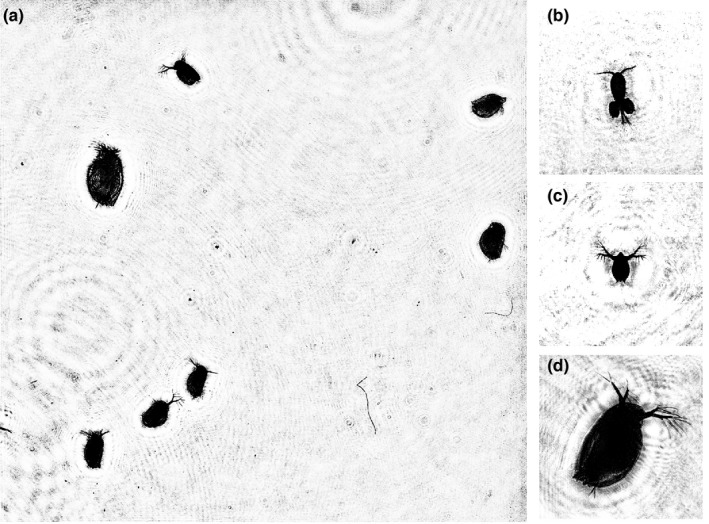
(a) 2D display of a holographic image of the studied volume with *Daphnia magna*. (b–d) Images of some plankton individuals reconstructed from digital holograms: b—*Cyclops vicinus*, c—*Daphnia pulex*, and d—*Daphnia magna*

The example of a 2D display of a holographic image obtained from a digital hologram recorded by a digital holographic camera is shown in Figure 2. A set of data obtained after digital hologram processing, on the basis of which the results of this study are presented, is available at Zenodo digital repository—http://dx.doi.org/10.5281/zenodo.4308667 (Dyomin, Morgalev, et al., 2020).

Statistical data analysis was performed using Statistica 10.

Since the distribution of variables did not always correspond to the normal law (Shapiro–Wilk's *W* test), and each experiment used a new assemblage of crustaceans, the assessment of differences and statistical significance of the result (*p*‐value) was carried out using a nonparametric Mann–Whitney *U* test.

## 3 RESULTS AND DISCUSSION

### 3.1 Phototropic response of crustacean

The preliminary series of experiments studied the time characteristics of the phototropic response of the *Daphnia magna* assemblage to attracting light. The experiment is partially described by us (Dyomin, Davydova, Morgalev, Olshukov, et al., 2019). According to holography data, chaotic movement with periodic daphnia release from the working volume was observed in the working volume of 1 L after the addition of zooplankton at the concentration of 3,000 ind m^−3^ (270 ind/cell). On average, 17.5 ± 3.4 *Daphnia* individuals passing through the working volume were recorded over 5 min without attracting light. The mean residence time of a *Daphnia* individual in the working volume was 5.23 ± 0.68 s.

After the inclusion of attracting light, the following concentration of *Daphnia* in the working volume was observed: From 120 to 150 *Daphnia* passed through the working volume over 5 min, however, due to the increased speed of movement, the residence time of a certain *Daphnia* in the working volume was slightly reduced to 4.25 ± 0.40 s. The time during which the *Daphnia* concentration in the working volume increased to 10 individuals ranged from 87 to 100 s. After the attracting light was turned off, the amount of *Daphnia* in the working volume began to decrease in 57.8 ± 5.3 s, and this process lasted approximately 10 min. After this time, the number of small crustaceans passing through the working volume decreased to a level corresponding to the level before the attracting light was on.

Based on these data, the following scheme for recording the measuring hologram set was chosen for further studies: Immediately after the attracting light is turned on, a pair of holograms is recorded with a time interval of 41.6 ms between them for subsequent calculation of the average speed of the zooplankton assemblage. This time interval is chosen based on the average speed of movement and the size of crustaceans—during this time, the displacement of each crustacean does not exceed the size of its body, that is, the images of the same particle reconstructed from adjacent holograms partially overlap. This makes it possible to definitely identify the paired images of the same particle in order to determine its speed and direction of movement. Further, such pairs were recorded every 30 s, 60 s, 90 s, 120 s, 150 s, 180 s, 210 s, 240 s, 270 s, and 300 s after the attracting light was turned on, that is, the recording interval was 5 min. To assess the background activity of crustaceans, registration was carried out before each illumination according to a similar scheme of a 5‐min interval without lighting.

**FIGURE 3:**
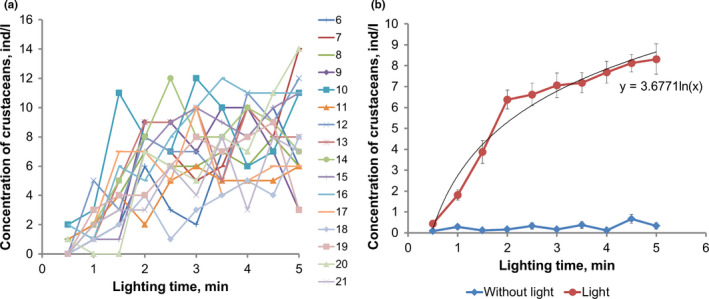
Phototropic responses of *Daphnia magna*. (a) Concentration of plankton individuals in the working volume in a pollutant‐free medium (lines of different color—time from the beginning of the experiment in hours). (b) Dynamics of the number of crustaceans in the working volume averaged over 21 hr, with and without lighting

The measuring hologram set was registered every hour throughout 21 hr of the experiment (Figure 3).

As stated above, after the attracting light was turned on, zooplankton naturally moved to the lighting zone and the concentration of individuals in the working volume increased. By the fifth minute of photostimulation, the average concentration of *Daphnia magna* was 8.3 ± 0.7 ind/L with the maximum value of 14 ind/L. There was almost no increase in the concentration without photostimulation (Figure 3b). The “concentration—lighting time” dependence differs from the linear one, so in Figure 3, the data are approximated by the logarithmic dependence. The concentration rate in these coordinates was equal to 3.67 ± 0.42 ind (L min)^−1^.

The phototropic response of the *Daphnia pulex* assemblage was weaker. The average concentration increase by the fifth minute of photostimulation was 5.2 ± 0.8 ind/L reaching the maximum of 12 ind/L. The concentration growth rate in the “concentration—lighting time” coordinates was 2.42 ± 0.19 ind (L min)^−1^.

The phototropic response of the *Cyclops* assemblage was almost absent. The average concentration by the fifth minute of photostimulation was 0.6 ± 0.2 ind (L min)^−1^ with the maximum value of 2 ind (L min)^−1^. The concentration growth rate in the “concentration—lighting time” coordinates was 0.16 ± 0.02 ind (L min)^−1^.

A mixed group of zooplankton species consisting of an equal number (90 each) of *Daphnia magna, Daphnia pulex,* and *Cyclops vicinus* showed a pronounced phototropic response. The average concentration by the fifth minute of photostimulation was 11.0 ± 1.5 ind (L min)^−1^ at the maximum increase of 20 ind (L min)^−1^. The concentration growth rate in the “concentration—lighting time” coordinates was 4.49 ± 0.29 ind (L min)^−1^. The pronounced phototropic response is associated with the prevalence of *Daphniidae* phytotrophes in the assemblage (180 of 270 individuals).

An attempt to isolate the mobility characteristics of different species from the group of species was not quite successful. First, as we have previously noted (Dyomin, Davydova, et al., 2020), the accuracy of taxonomic affiliation of objects reconstructed from a hologram is not high: Copepoda—86 ± 9% and Cladocera—77 ± 2%. Second, the conjugation of the recognition algorithm with the hologram reconstruction algorithm requires quite large computational resources and did not allow obtaining behavioral response characteristics in dynamics in our experiment. Therefore, the contribution of crustaceans of different species to general reactivity was studied in the experiments with monocultures.

It should be noted that the response of zooplankton assemblage formed from crustaceans of older age (5–7 days) may vary quantitatively, but the overall responsiveness ratio remains the same: Concentration growth rates are maximum for the mixed group of species (5.95 ± 0.38 ind (L min)^−1^) and decrease for *Daphnia magna* and *Cyclops vicinus* (3.54 ± 0.33 ind (L min)^−1^ and 0.05 ± 0.02 ind (L min)^−1^, respectively).

### 3.2 Phototropic response of crustacean in the presence of potassium bichromate

A solution of potassium bichromate at the concentrations of 0.06 mg/L and 0.12 mg/L was used as a model pollutant. The toxicity of the pollutant by mortality and reduction of daphnia mobility was determined in accordance with (ISO, 2012).

Toxicity is generally estimated to be LC_50_ (50% lethal concentration), which is the amount of a substance dissolved in water required to kill 50% of test animals during a predetermined observation period. EC_50_ (half‐maximum effective concentration) is used to assess nonlethal effects on physiological and behavioral functions. This is the concentration of a substance causing an effect equal to half the maximum possible for a given substance after a certain period of time.

The earlier studies showed that the toxic effect on the behavioral responses of *Daphnia magna* consisting in a change in the nature of movement was observed from the concentration of 0.011 ± 0.001 mg/L, and a 50% response change (EC_50_) was observed at a pollutant concentration of 0.15 ± 0.02 mg/L (Dyomin, Gribenyukov, Davydova, et al., 2019).

The sensitivity of *Cyclops vicinus* to potassium bichromate is significantly lower. The study showed that the LC_50,_ therefore, was 29.6 ± 9.6 mg/L. This major difference in sensitivity is caused by the fact that *Cyclops* are not filtration organisms. Similar data are obtained by other researchers (Noskov, 2011).

#### 3.2.1 Behavioral response of the mixed group of crustacean species

**FIGURE 4:**
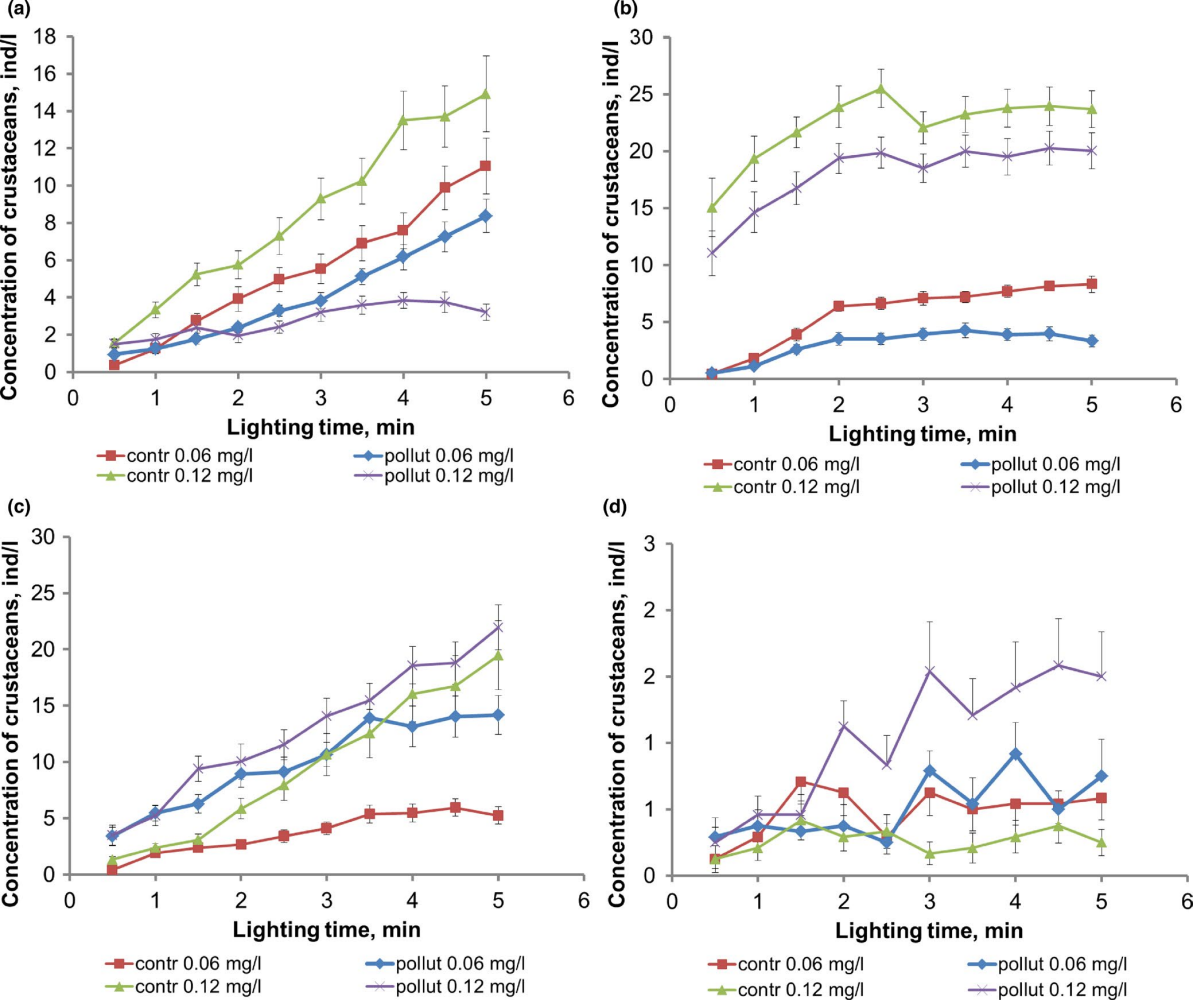
Change in the average phototropic response of the mixed group of *Crustacea* (a), *Daphnia magna* (b), *Daphnia pulex* (c), and *Cyclops vicinus* (d) when introducing potassium bichromate

The toxicant at the concentration of 0.06 mg/L caused a decrease in the severity of the phototropic response of the mixed group of zooplankton species (Figure 4a): The concentration growth rate in the working volume decreased from 4.49 ± 0.29 ind (L min)^−1^ to 3.15 ± 0.22 ind (L min)^−1^ (*p* = .0013). This fact may be explained by the devastating effect of the toxicant on the nervous system of zooplankton and, hence, a decrease in its mobility.

As the toxicant concentration increased to 0.12 mg/L, the inhibition of the phototropic response of the mixed group of species was increased: The concentration growth rate in the working volume decreased from 5.95 ± 0.38 ind (L min)^−1^ to 1.05 ± 0.11 ind (L min)^−1^ (*p* = .0013) amounting to 17.6% of the background value compared to 70.0% when the toxicant concentration was 0.06 mg/L.

#### 3.2.2 Behavioral response of *Daphnia magna*

The pollutant concentration of 0.06 mg/L results in a pronounced inhibition of the phototropic response of *Daphnia magna* (Figure 4b): The concentration growth rate in the working volume decreased from 3.67 ± 0.42 ind (L min)^−1^ to 1.59 ± 0.31 ind (L min)^−1^ (*p* = .0003).

At a higher bichromate concentration of 0.12 mg/L, there was no reliable change in the concentration growth rate compared to the control situation without a pollutant—from 3.54 ± 0.33 ind (L min)^−1^ to 3. 9 ± 0.26 ind (L min)^−1^ (*p* > .05).

#### 3.2.3 Behavioral response of *Daphnia pulex*

There was a paradoxical response of *Daphnia pulex*—a critical increase in the phototropic response of this group of species (Figure 4c): The concentration growth rate increased from 2.42 ± 0.19 ind (L min)^−1^ to 5.17 ± 0.46 ind (L min)^−1^ (*p* = .0001).

A slight decrease in the phototropic response was observed at the concentration of 0.12 mg/L: The concentration growth rate decreased from 8.14 ± 0.80 ind (L min)^−1^ to 7.86 ± 0.69 ind (L min)^−1^ (*p* = .40). The tendency to inhibit the phototropic response at a higher toxicant concentration than that of *Daphnia magna* may be caused by the fact that the size of the body, and therefore, the amount of fluid to be filtered is smaller for *Daphnia pulex*.

#### 3.2.4 Behavioral response of *Cyclops vicinus*

As expected, there was no reliable change in the behavior of *Cyclops vicinus* when potassium bichromate was introduced at the concentration of 0.06 mg/L (Figure 4d): The concentration growth rate in the working volume changed unreliably from 0.16 ± 0.02 ind (L min)^−1^ to 0.21 ± 0.03 ind (L min)^−1^ (*p* = .11).

By contrast, the pollutant concentration of 0.12 mg/L leads to an intense yield of crustaceans into the working volume. At an initially low concentration in the attracting light zone (0.2 ± 0.1 ind/L) and in the presence of toxicants, their concentration increases to 1.5 ± 0.3 ind/L. Accordingly, the concentration growth rate in the working volume increases from 0.05 ± 0.02 ind (L min)^−1^ to 0.63 ± 0.12 ind (L min)^−1^ (*p* = .0001). This may be associated with the avoidance response when high toxicity of the medium is detected.

Thus, the behavioral response of the mixed group of crustaceans consists of somewhat divergent changes in responsiveness. Under the conditions of our experiment, the *Daphnia magna* responses were critical, but with a change in the ratio of species it is possible to implement other response variants of the natural group of species.

### 3.3 Possible reasons for differences in responses with a toxicant

The most pronounced pattern of zooplankton response to a toxicant is inhibition of phototropic response—concentration of crustaceans in the attracting light zone. One mechanism for such changes may be the direct effect of the toxicant on the nervous system of crustaceans thus leading to a change in their behavior (Dallakyan et al., 2017; Kikuchi et al., 2017).

This may be additionally confirmed by the obtained data on the change in the swimming activity of crustaceans in the presence of potassium bichromate. Ten *Daphnia magna* crustaceans were placed into 1‐L laboratory glass cups with a cultivation medium, and their distribution by column height was recorded (15 cm from the liquid surface to the bottom). Potassium bichromate was added to the test containers up to the concentration of 0.03 mg/L; 0.06 mg/L; 0.12 mg/L; 0.32 mg/L, and 0.56 mg/L. The behavioral responses were registered with the counting of dead crustaceans every 5, 15, 30, 60 min, 24 hr, and 48 hr of the experiment. The experiment was repeated three times.

**FIGURE 5:**
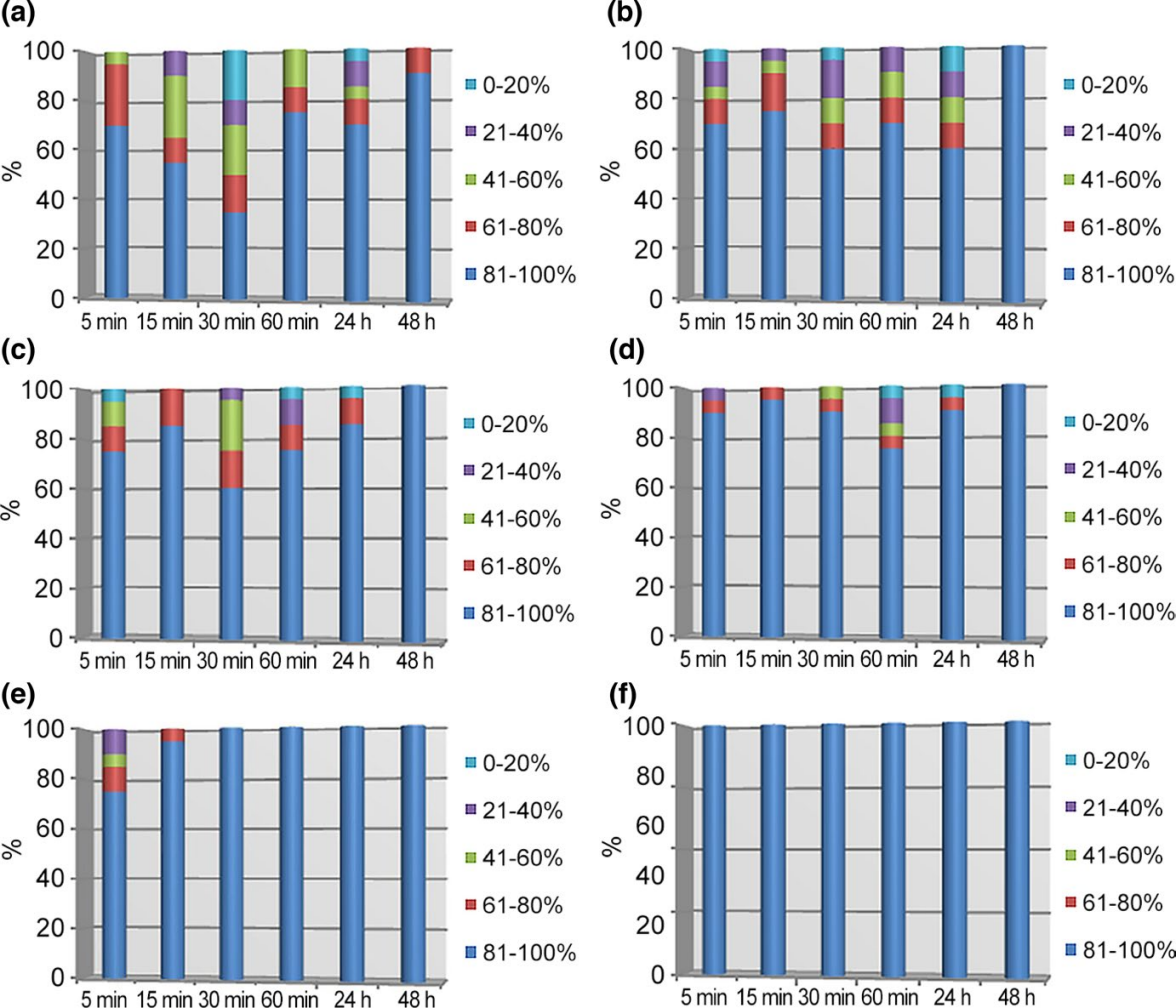
Dynamics of crustaceans vertical distribution during medium pollution with different concentrations of potassium bichromate. The color indicates water layers at different depths from the liquid surface (bottom—100%). Horizontal axis—time from the beginning of the experiment. Vertical axis—share (%) of crustaceans in the corresponding layer. (a) Control group, without a pollutant, (b) concentration of potassium bichromate—0.03 mg/L, (c) 0.06 mg/L, (d) 0.12 mg/L, (e) 0.32 mg/L, (f) 0.56 mg/L

In the control series, during all the observation periods the movement of crustaceans corresponded to the norm: Daphnia were active, moved stepwise, “floated” up and down. The distribution of crustaceans by the volume of the cultivation medium is shown in Figure 5a.

In the first five minutes, the daphnia were in the bottom half of the container gradually rising into the upper layers, and in 30 min of the experiment, they were evenly distributed throughout the entire volume of the cup. In 24 hr, 70% of daphnia moved to the bottom of the container. In 48 hr, 90% of individuals actively moved along the bottom of the cup.

After introducing a toxicant at the concentration of 0.03 mg/L into the cultivation medium for 24 hr, the distribution of daphnia over the volume of the test medium was almost the same as in the control series (Figure 5b). Approximately 70% of individuals were evenly distributed in the bottom layer. There were no deviations in the nature of movement of crustaceans. Daphnia died in 48 hr after the introduction of the pollutant, after which mortality rate amounted to 36.6%, and the survived individuals moved along the bottom of the container.

The depth distribution of crustaceans at the concentration of 0.06 mg/L and 0.12 mg/L is shown in Figure 5c,d. As the concentration of the pollutant increased, the number of individuals rising into the upper layers of the medium decreased, which indicates an inhibition of their motor activity. In 24 hr, 10% of individuals turned over the head and 10% and 15% of animals died at a bichromate concentration of 0.06 mg/L and 0.12 mg/L, respectively. In 48 hr of observation, 47.2% of crustaceans died in a medium with a potassium bichromate concentration of 0.06 mg/L and 52.4% of daphnia in a medium with a concentration of 0.12 mg/L. The survived daphnia slowly moved along the bottom of the container.

When a pollutant at the concentration of 0.32 mg/L was introduced into the cultivation medium for up to 15 min, single individuals rose to the upper layers of the medium; during all other periods of the experiment, daphnia evenly moved along the bottom of the cup (Figure 5e). The death rate of daphnia in 24 and 48 hr of observation was 35.0% and 69.2%, respectively.

When the water medium was contaminated with a pollutant at the concentration of 0.56 mg/L, 100% of animals were found at the bottom of the cup at all times of the experiment (Figure 5f). Until 60 min, daphnia moved calmly, stepwise. In 60 min, the inhibition of the motor activity of animals and turns over the head were noted. The death rate of daphnia in 24 hr was 35.0%, in 48 hr—73.4%.

Thus, one of the mechanisms of subsequent inhibition of the phototropic response is the decrease in motor activity, accumulation at the bottom of the container, and a partial reduction of the number of crustaceans as a result of their death.

Phasal nature of the swimming change deserves special attention. The periods of increased swimming activity are recorded even at bichromate concentrations of 0.06 mg/L and 0.12 mg/L.

The action of the altering factor of sublethal intensity causes the dynamics of states typical for the development of nonspecific adaptation syndrome: warning stage (stimulation, mobilization of adaptation abilities), resistance stage (stability), exhaustion stage (Selye, 1946). These factors have been repeatedly confirmed both for certain different organisms and entire populations (Patin, 2004).

The most rapid and relatively easily recorded effects occur at physiological, biochemical, and organizational levels.

Stimulation, that is, intensification of vital functions, is the first response phase. The following externally noted resistance does not confirm the lack of response: Tolerance at the organizational level may hide deep intracellular and molecular processes, slowly accumulating changes in cells, their organelles, chromosomes, DNA, and other microstructures. The prolonged effect or an increase in toxicant concentration leads to depression, that is, suppression of vital activity, which may ultimately lead to death.

These processes may explain the lower suppression of *Daphnia magna* phototropic response at a higher toxicant concentration (by 60% at 0.06 mg/L and by 16% at 0.12 mg/L) due to decreased sensitivity at the beginning of the exhaustion phase, as well as the initially inverted responsiveness of *Daphnia pulex* that decreases as the toxicant concentration increases.

It may be assumed that the concentrations causing the phase of tolerance and suppression for *Daphnia magna* are stimulating for *Daphnia pulex* due to their reduced sensitivity. This is due to the fact that the size of the body, and therefore the amount of fluid to be filtered, is less.

**FIGURE 6:**
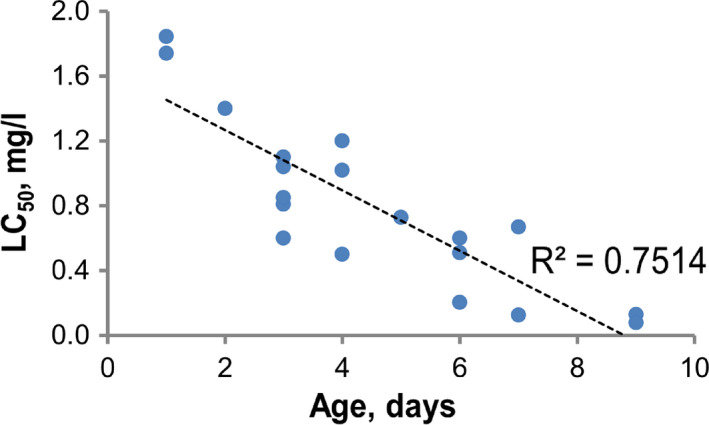
Change in sensitivity of *Daphnia pulex* (LC_50_) to the introduction of potassium bichromate with increasing age of individuals

The increase of the sensitivity of *Daphnia* as age increases, and accordingly body sizes, is described in Traudt et al. (2017) and studied by us in the survival test (LC_50_) of *Daphnia pulex* (Figure 6). As the age of crustaceans increases, the toxicant concentration leading to the death of 50% individuals (LC_50_) decreases from 1.84 mg/L for 1‐day individuals to 0.13 mg/L for 9‐day individuals, that is, the sensitivity to this toxicant increases by more than an order of magnitude.

Other reasons may explain the differences in *Cyclops vicinus* responses. *Cyclops* are mainly zootrophes and feed on protozoa, rotifers, small crustaceans. In this regard, light with a wavelength corresponding to the reflection spectrum of chlorophyll does not seem to be a signal of food for them and, accordingly, an attractor.

The phototropic response of *Cyclops vicinus* increasing with an increase in toxicant concentration may be associated with the avoidance response at high toxicity of the medium and active movement to a “safe” zone with chlorophyll‐containing microorganisms.

## 4 CONCLUSION

The phototropic response of the model *Crustacea* assemblage containing *Daphnia magna*, *Daphnia pulex,* and *Cyclopidae* in equal amounts is not a simple superposition of phototropic responses of the species included in it. The response of the group of species to toxicant application expressed as a decrease in the phototropic response exceeds the sensitivity of each of the species, and the inhibition of the phototropic response monotonically depends on toxicant concentration.

The performed studies showed the perspectiveness of using the phototropic response of zooplankton to monitor the quality of its habitat for early detection of water pollution. The established dependence of the phototropic response of plankton individuals on their taxonomic affiliation indicates the need for parallel dynamic definition of the ratio between phyto‐ and zootrophic species of autochthonous plankton in the monitoring zone.

The possibility of this definition for the early detection of water pollution in the continuous monitoring mode is ensured by submersible digital holographic cameras (DHC) and hydrobiological probes developed at Tomsk State University.

## CONFLICT OF INTEREST

The authors claim no conflict of interest.

## AUTHOR CONTRIBUTIONS

**Victor Dyomin:** Conceptualization (lead); funding acquisition (lead); methodology (equal); supervision (lead); writing‐review & editing (lead). **Yuri Morgalev:** Conceptualization (lead); data curation (lead); investigation (equal); methodology (equal); writing‐original draft (lead); writing‐review & editing (equal). **Igor Polovtsev:** Conceptualization (lead); investigation (equal); methodology (equal); writing‐review & editing (equal). **Alexandra Davydova:** Data curation (lead); software (lead); writing‐review & editing (equal). **Sergey Morgalev:** Investigation (lead); writing‐review & editing (equal). **Nikolay Kirillov:** Software (lead); writing‐review & editing (equal). **Tamara Morgaleva:** Investigation (lead); writing‐review & editing (equal). **Alexey Olshukov:** Software (lead); writing‐review & editing (equal).

## Data Availability

The data set is available at the digital repository Zenodo with https://doi.org/10.5281/zenodo.4308667 (Dyomin, Morgalev, et al., 2020).
